# Comparative Analysis of Putative Prognostic and Predictive Markers in Neuroblastomas: High Expression of PBX1 Is Associated With a Poor Response to Induction Therapy

**DOI:** 10.3389/fonc.2019.01221

**Published:** 2019-11-15

**Authors:** Renata Veselska, Marta Jezova, Michal Kyr, Pavel Mazanek, Petr Chlapek, Viera Dobrotkova, Jaroslav Sterba

**Affiliations:** ^1^Laboratory of Tumor Biology, Department of Experimental Biology, Faculty of Science, Masaryk University, Brno, Czechia; ^2^Department of Pediatric Oncology, Faculty of Medicine, University Hospital Brno, Masaryk University, Brno, Czechia; ^3^International Clinical Research Center, St. Anne's University Hospital Brno, Brno, Czechia; ^4^Department of Pathology, Faculty of Medicine, University Hospital Brno, Masaryk University, Brno, Czechia

**Keywords:** neuroblastoma, prognostic markers, predictive markers, PBX1, NF1, HOXC9, immunohistochemistry

## Abstract

The survival rate for patients with high-risk neuroblastomas remains poor despite new improvements in available therapeutic modalities. A detailed understanding of the mechanisms underlying clinical responses to multimodal treatment is one of the important aspects that may provide precision in the prediction of a patient's clinical outcome. Our study was designed as a detailed comparative analysis of five selected proteins (DDX39A, HMGA1, HOXC9, NF1, and PBX1) in one cohort of patients using the same methodical approaches. These proteins were already reported separately as related to the resistance or sensitivity to retinoids and as useful prognostic markers of survival probability. In the cohort of 19 patients suffering from high-risk neuroblastomas, we analyzed initial immunohistochemistry samples obtained by diagnostic biopsy and post-induction samples taken after the end of induction therapy. The expression of DDX39A, HMGA1, HOXC9, and NF1 showed varied patterns with almost no differences between responders and non-responders. Nevertheless, we found very interesting results for PBX1: non-responders had significantly higher expression levels of this protein in the initial tumor samples when compared with responders; this expression pattern changed inversely in the post-induction samples, and this change was also statistically significant. Moreover, our results from survival analyses reveal the prognostic value of PBX1, NF1, and HOXC9 expression in neuroblastoma tissue. In addition to the prognostic importance of PBX1, NF1, and HOXC9 proteins, our results demonstrated that PBX1 could be used for the prediction of the clinical response to induction chemotherapy in patients suffering from high-risk neuroblastoma.

## Introduction

Neuroblastoma (NBL) is the most common extracranial solid tumor in children, accounting for 6–8% of all childhood cancers and more than 10% of pediatric cancer-related mortality. NBL is a complex and heterogeneous disease with several factors determining the clinical outcome, especially the age at diagnosis, stage of the disease (localized vs. metastasizing), and biological features of the tumor (MYCN copy number determined by fluorescence *in situ* hybridization, DNA content measured by flow cytometry, and tumor histology evaluated using the International NBL Pathology Classification system). Based upon these factors, NBL is classified into low-, intermediate-, or high-risk categories. The estimated risk category correlates with the clinical outcome of the disease: patients with low-risk or intermediate-risk NBL have a 5-year overall survival rate exceeding 90%, whereas this value is ~40% for patients suffering from high-risk NBL (1, 2).

The stratification of patients into the risk categories described above represents a key step in choosing the right therapy for the right patient. Children with biologically favorable non-metastatic NBL generally require little or no cytotoxic therapy. In contrast, outcomes for patients with high-risk NBL remain poor despite new improvements of available therapeutic modalities, including biological therapy with differentiation inducers and immunotherapy with chimeric monoclonal antibodies (2–4).

Standard chemotherapy for high-risk NBL includes dose-dense or dose-intensive myeloablative regimens using alkylating agents, platinum compounds, topoisomerase-II inhibitors (doxorubicin, etoposide), and topoisomerase-I inhibitors (topotecan, irinotecan) followed by autologous hematopoietic stem cell transplantation (4, 5). At the end of this intensive multimodal treatment, the administration of retinoids in patients with minimal residual disease was shown to be effective and able to delay or prevent tumor relapse after myeloablative therapy (4, 6, 7). Nevertheless, even though retinoids are able to improve the survival of patients with high-risk NBL, ~50% of these patients were resistant to this treatment or developed resistance during therapy (8).

A detailed understanding of the mechanisms underlying the response of NBL to multimodal treatment is one of the important aspects that may provide precision in the prediction of a patient's clinical outcome, especially within the group of high-risk NBL. In this regard, resistance or sensitivity to retinoids is one of the discussed aspects of this strategy (8). A number of potential molecular mechanisms of resistance to retinoid therapy have been described over the past decade (9). Detailed investigation of the mechanisms of resistance to retinoids led to the identification of several molecules that are discussed as possible predictive biomarkers of clinical response to the treatment with retinoids (10).

In various types of tumor cells, including NBL, several key mediators of retinoid action were recently identified: NF1, HOXC9, or PBX1 (11–13). Based on published results, these proteins can be successfully used for the identification of NBL cell lines showing resistance to retinoids under *in vitro* conditions. Interestingly, certain studies have suggested that some of these molecules could also be used as prognostic markers for estimating survival probability in clinical practice (11, 13, 14).

Nevertheless, the clinical outcome of patients suffering from high-risk NBL is influenced by many other factors, including simple resistance or sensitivity to retinoids administered at the end of the intensive multimodal treatment. To elucidate the actual usefulness of these putative markers in clinical practice, our present study aimed to thoroughly analyze five selected markers already reported to be related to retinoid action (10): DDX39A, HMGA1, HOXC9, NF1, and PBX1. We analyzed the expression of these proteins by immunohistochemistry (IHC) in one cohort of patients suffering from high-risk NBL who underwent intensive induction chemotherapy and were finally treated with retinoids. This unique design allowed us to compare the reliability of these markers for the prediction of therapeutic response if their expression is related to the same set of clinical data. Finally, we also performed survival probability analyses in relation to the expression of these five protein markers to evaluate their prognostic usefulness using the same cohort of patients.

## Materials and Methods

### Tumor Samples

Nineteen samples of newly diagnosed, untreated, high-risk NBL (11 male patients, eight female patients; age range at the time of diagnosis, 19 months−12-years old) were included in this study. In addition to these samples from initial biopsies, we also analyzed an additional 12 samples taken from the same patients after intensive induction chemotherapy. Formalin-fixed, paraffin-embedded (FFPE) tumor samples were retrieved from the files of the Department of Pathology, University Hospital Brno, Czech Republic. Written informed consent was obtained from each participant or his/her legal guardian before entering into this study. The Research Ethics Committee of the School of Science, Masaryk University (Brno, Czech Republic) approved the study protocol.

### Immunohistochemistry

Representative sections from archival FFPE tumor samples were selected by one experienced pathologist (MJ) and processed for IHC as described previously in detail (15). All antibodies used in this study are specified in Table 1. In each IHC experiment, positive and negative controls were also evaluated (Supplementary Figure 1): tissues used as positive controls are also described in Table 1, and negative controls were processed without the primary antibodies. For each of the evaluated protein markers, specific nuclear or cytoplasmic immunostaining (as specified in the Table 1) was considered positive. The slides were evaluated with an Olympus BX50 light microscope at ×200 magnification. At least five discrete foci of tumor tissue were analyzed per sample by the same experienced pathologist (MJ), and the average staining intensity and the percentage of antigen-positive cells were determined. The percentage of antigen-positive tumor cells (TC) was categorized into five levels: – (0% positive TC), +/– (1–10% positive TC), + (11–50% positive TC), ++ (51–80% positive TC), and +++ (81–100% positive TC). The intensity of immunostaining (immunoreactivity, IR) was classified as none (0), weak (1), medium (2), or strong (3). Finally, the total immunoscores were calculated for individual antigens by multiplying the median percentage category of positive cells by their respective immunoreactivity as described previously (16) with possible immunoscore values ranging from 0 to 300.

**Table 1:** Antibodies and positive controls used in this study. N, nuclear staining; C, cytoplasmic staining.

**Antigen**	**Type/Host**	**Clone**	**Manufacturer**	**Dilution**	**Positive control**
DDX39A	Monoclonal/rabbit	EPR13508	Abcam	1:150	Human testis (N)
HMGA1	Monoclonal/rabbit	D1A7	Cell Signaling	1:500	Human colorectal cancer (N)
HOXC9	Polyclonal/rabbit	–	Bioss	1:100	Human kidney (N, C)
NF1	Polyclonal/rabbit	–	Santa Cruz Biotechnology	1:50	Human pancreas (C)
PBX1	Monoclonal/mouse	4A2	LSBio	1:50	Human pancreas (N)

### Statistical Analysis

IR and the percentage of IHC-stained TC were analyzed separately on a semiquantitative ordinal scale for baseline tissue samples. Proportions of patients with particular immunoscores are shown in bar plots, and differences between responders and non-responders were tested using the chi-square test. Summary statistics and raw data are presented in combined dot and box plots for baseline tissue samples. Differences between responders and non-responders were tested using the Mann-Whitney test. Immunoscores were also calculated for tissue samples after induction therapy, and pre-posttreatment differences in immunoscores between responders and non-responders were evaluated using factor ANOVA and displayed in error bar plots. The clinical significance of immunoscores was evaluated using survival analysis. For statistical purposes, data were dichotomized into low- and high-expression groups based on the median values of each particular parameter. Kaplan–Meier curves were plotted for event-free survival (EFS) and overall survival (OS), and differences between low- and high-expression groups were tested using log-rank tests. Analyses were performed using R software version 3.5.1. (17), and alpha = 0.05 was considered significant. We report raw *p*-values without correction for multiple testing because all tests we made are reported. Corrected *p*-values may thus be computed using a method according to the reader's selection. Nevertheless, we rather discourage from routine performing usual corrections. Our results are prone to the risk of overcorrection due to correlated measures, already preselected set of putative markers, low power of tests (in general) for categorical data, and the purpose of the study. We are interested in any indicator of possible predictive and/or prognostic markers, and we would rather not inflate the type II error.

## Results

### Cohort Description and Expression Patterns of Evaluated Proteins

**Figure 1:**
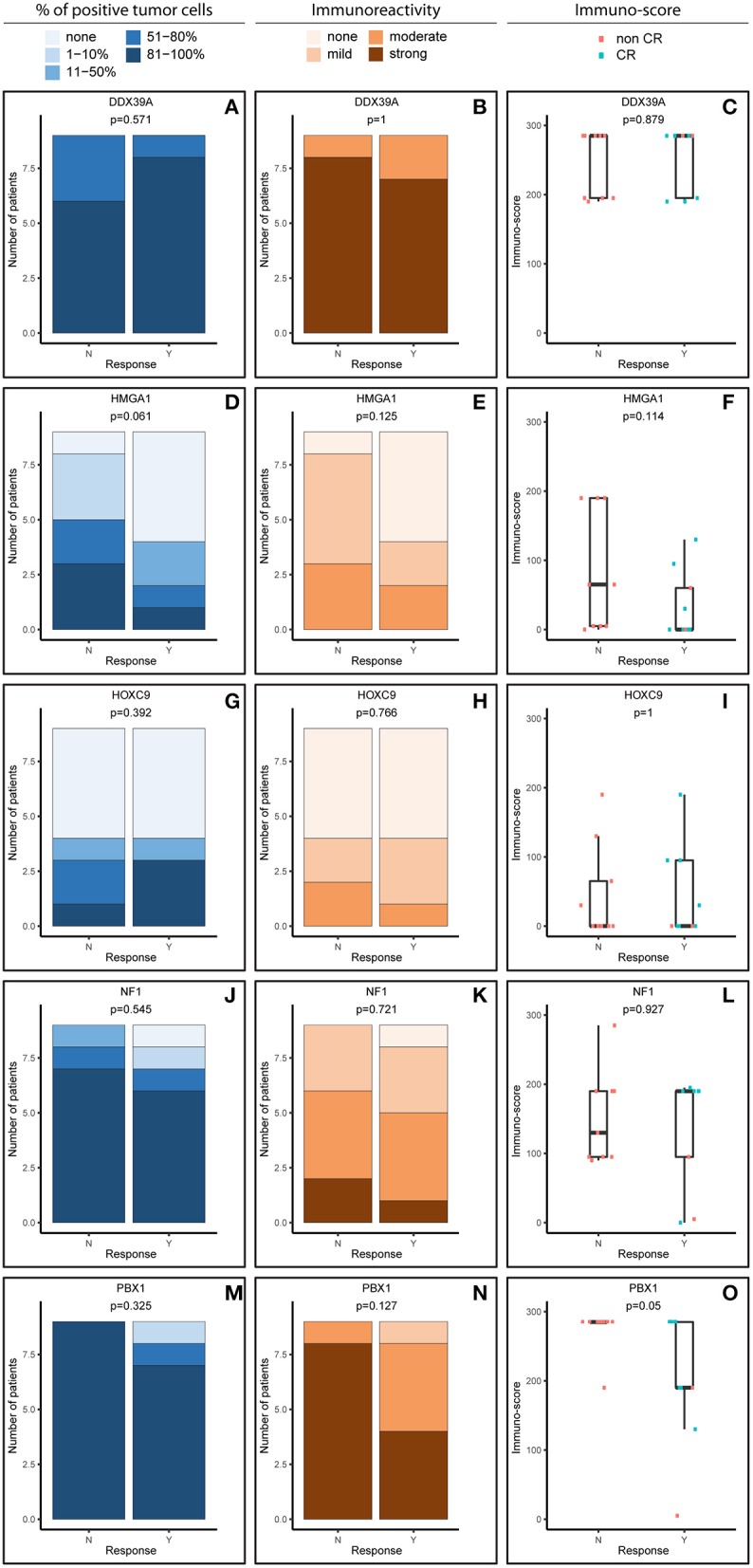
Comparative immunohistochemistry (IHC) analysis of DDX39A **(A–C)**, HMGA1 **(D–F)**, HOXC9 **(G–I)**, NF1 **(J–L)**, and PBX1 **(M–O)** in the initial samples. Immunoscores were calculated for individual antigens by multiplying the median percentage category of positive cells by their respective immunoreactivity. N, non-responder; Y, responder. CR, complete remission.

**Figure 2:**
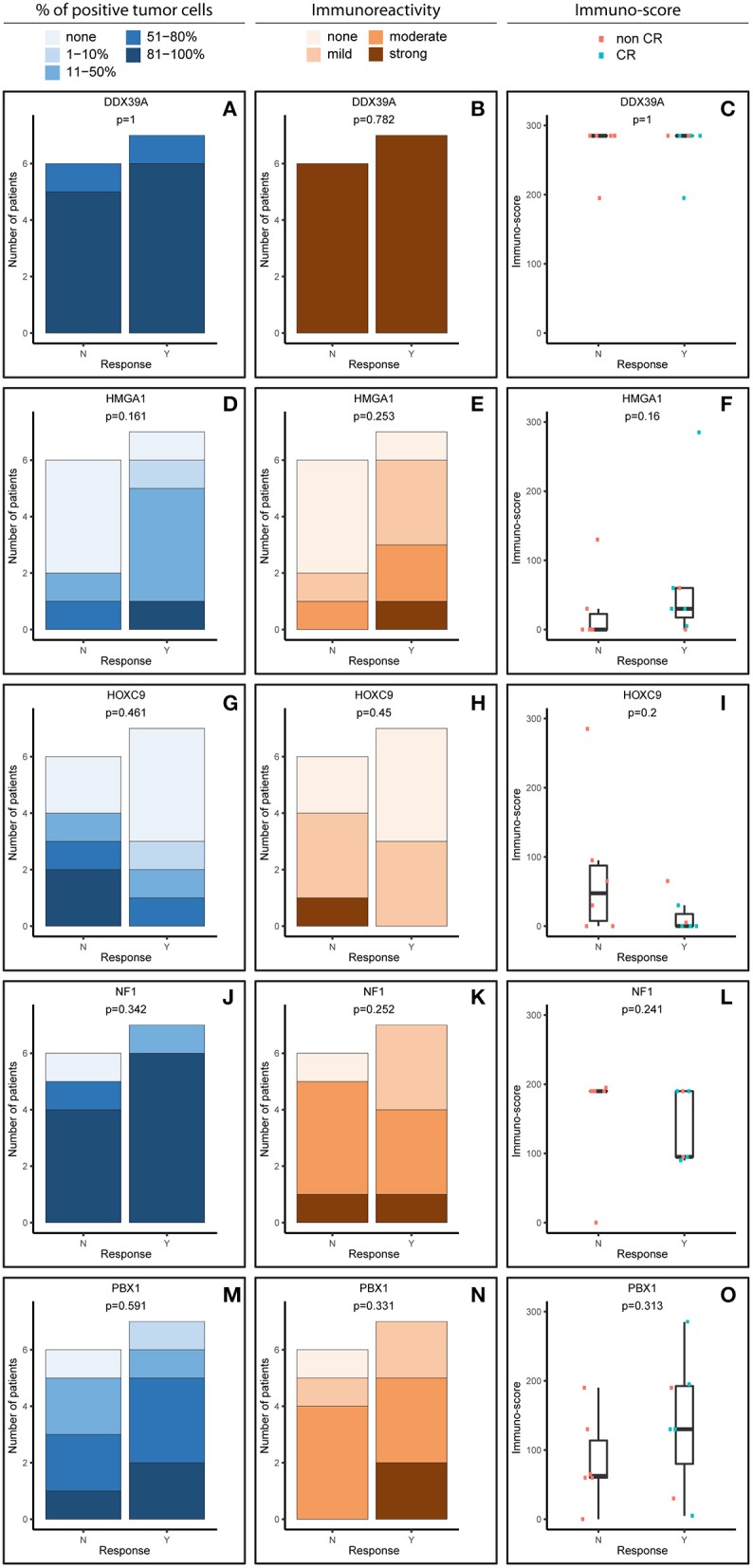
Comparative immunohistochemistry (IHC) analysis of DDX39A **(A–C)**, HMGA1 **(D–F)**, HOXC9 **(G–I)**, NF1 **(J–L)**, and PBX1 **(M–O)** in the post-induction samples. Immunoscores were calculated for individual antigens by multiplying the median percentage category of positive cells by their respective immunoreactivity. N, non-responder; Y, responder. CR, complete remission.

A cohort consisting of 19 patients suffering from high-risk NBL was included in this study: a detailed clinical description of these patients is given in the Table 2. All of them were treated according the same Children's Oncology Group ANBL 0532 protocol. In this cohort, we analyzed two sets of FFPE tumor samples using IHC: (i) initial samples obtained by a diagnostic biopsy (Figure 1) and (ii) post-induction samples taken after the end of induction therapy (Figure 2). Although all patients were originally chosen for this cohort according to the availability of both FFPE samples—initial and post-induction—some samples had to be omitted from the final analyses due to poor quality. The initial sample obtained from patient no. 5 and the post-induction samples from patients nos. 1, 2, 6, 8, 11, and 19 were excluded from this cohort. In total, 18 initial samples and 13 post-induction samples were ultimately included in the statistical analyses. Complete detailed results are given in the Table 3.

**Table 2:** Clinical description of the patients included in this study.

**Patient no**.	**Age range (months)**	**Tumor histology**	**INSS stage**	**MYCN status**	**Response to the induction therapy**	**Status**
1	30–35	UH	3	Amp	Y	NED
2	30–35	UH	4	Neg	Y	NED
3	30–35	UH	4	Neg	N	DOD
4	40–45	UH	4	Neg	Y	NED
5	46–50	N/A	4	Neg	Y	NED
6	15–20	UH	2B	Amp	Y	NED
7	145–150	UH	4	Neg	Y	NED
8	30–35	UH	4	Amp	N	DOD
9	15–20	FH	4	Neg	N	NED
10	30–35	UH	4	Amp	N	AWD
11	10–15	UH	4	Neg	N	DOD
12	60–65	UH	4	Neg	Y	NED
13	20–25	UH	4	Amp	N	AWD
14	26–30	UH	4	Amp	N	NED
15	20–25	UH	4	Amp	Y	NED
16	40–45	N/A	4	N/A	Y	AWD
17	46–50	UH	4	Neg	N	AWD
18	26–30	UH	4	Amp	Y	DOD
19	100–105	UH	4	Neg	N	DOD

*Age range at the time of diagnosis (in months). Tumor histology according INPC (Shimada system): UH, unfavorable histology; FH, favorable histology; N/A, not available. INSS stage according to the International Neuroblastoma Staging System Committee (INSS) system. Response to the induction therapy: Y, yes = responder (partial remission or better); N, no = non-responder (stable disease or worse). Status: NED, no evidence of disease; DOD, dead of disease; AWD, alive with disease*.

**Table 3:** Results of IHC analyses of DDX39A, HMGA1, HOXC9, NF1, and PBX1 expression.

**Sample no**.	**Sample type**	**DDX39A**		**HMGA1**		**HOXC9**		**NF1**		**PBX1**	
		**% TC**	**IR**	**% TC**	**IR**	**% TC**	**IR**	**% TC**	**IR**	**% TC**	**IR**
1a	I	+++	3	–	0	–	0	–	0	+++	3
2a	I	+++	2	–	0	–	0	+++	1	+++	2
3a	I	+++	3	+/–	1	++	1	+++	1	+++	3
3b	P	+++	3	–	0	–	0	+++	2	++	1
4a	I	+++	3	–	0	–	0	+++	1	+++	2
4b	P	+++	3	–	0	++	1	+++	2	+++	2
5a	P	+++	3	+++	3	–	0	+++	1	+/–	1
6a	I	+++	3	–	0	+++	2	+++	2	++	2
7a	I	++	3	+	1	+++	1	+++	2	+++	2
7b	P	+++	3	+/–	1	+	1	+	3	++	2
8a	I	+++	3	++	1	–	0	+++	2	+++	3
9a	I	+++	3	++	1	++	2	+++	1	+++	3
9b	P	+++	3	+	1	++	1	+++	2	++	2
10a	I	+++	3	+/–	1	+	1	+++	3	+++	3
10b	P	++	3	–	0	+	1	+++	2	+	2
11a	I	+++	2	+/–	1	–	0	+++	2	+++	3
12a	I	+++	2	–	0	+++	1	+++	2	+++	3
12b	P	+++	3	+	1	–	0	+++	2	++	3
13a	I	++	3	–	0	+++	2	++	2	+++	3
13b	P	+++	3	–	0	+++	1	++	3	+++	2
14a	I	++	3	+++	2	–	0	+++	1	+++	3
14b	P	+++	3	–	0	–	0	–	0	–	0
15a	I	+++	3	++	2	+	1	++	3	+++	3
15b	P	++	3	+	1	–	0	+++	1	++	2
16a	I	+++	3	+	2	–	0	+/–	1	+/–	1
16b	P	+++	3	+	2	+/–	1	+++	1	+	1
17a	I	++	3	+++	2	–	0	+	3	+++	2
17b	P	+++	3	++	2	+++	3	+++	2	+	2
18a	I	+++	3	+++	1	–	0	+++	2	+++	3
18b	P	+++	3	+	2	–	0	+++	2	+++	3
19a	I	+++	3	+++	2	–	0	+++	2	+++	3

*The percentage of antigen-positive tumor cells (TC) was counted and categorized into five levels: – (0% positive TC), +/– (1–10% positive TC), + (11–50% positive TC), ++ (51–80% positive TC), and +++ (81–100% positive TC). The intensity of immunostaining (immunoreactivity, IR) was classified as none (0), weak (1), medium (2), or strong (3)*.

For analysis purposes, the patients were subdivided into two groups: responders achieving at least partial remission and non-responders with stable disease or worse outcome (Figures 1, 2). The response definition is based on the International Neuroblastoma Risk Group response criteria, and it was evaluated as overall response, i.e., combination of primary tumor response and response of metastatic sites. For each sample and protein marker, the percentage of positive TC (Figures 1A,D,G,J,M, 2A,D,G,J,M) as well as the IR (Figures 1B,E,H,K,N, 2B,E,H,K,N) was evaluated. In the next step, immunoscore values were determined for each sample (Figures 1C,F,I,L,O, 2C,F,I,L,O).

In general, we observed several obvious differences in the expression patterns among these five proteins in the initial samples. DDX39A (Figures 1A–C) and PBX1 (Figures 1M–O) exhibited the highest proportions of positive TC in the sample and the highest IR in both responders and non-responders, which also led to the highest immunoscore values. High proportions of NF1-positive tumor cells were also found, but the IR was almost moderate to mild for this protein (Figures 1J–L). Moderate to mild IR was also observed for HMGA1 (Figures 1D–F) and HOXC9 (Figures 1G–I), and the percentage of cells positive for these markers and their respective immunoscores were reduced compared with the previously mentioned proteins.

### Analysis of Expression Patterns of Evaluated Proteins in Relation to the Response to Induction Chemotherapy

**Figure 3:**
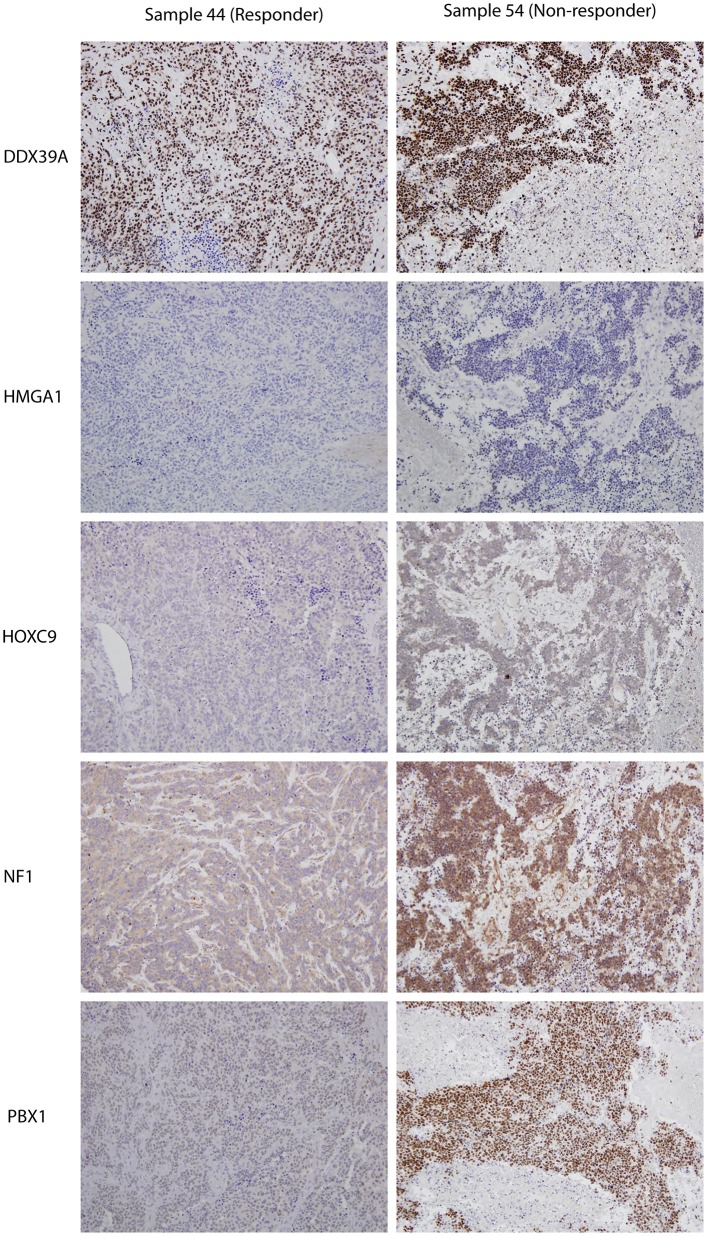
Representative expressions of DDX39A, HMGA1, HOXC9, NF1, and PBX1 in responder (sample 7a) and non-responder (sample 10a) initial tumor samples. Original magnification, 200×.

Although the analyzed markers exhibited varied expressions in the initial samples, nearly no significant changes were detected in responders and non-responders. Nevertheless, the most interesting result was found for PBX1: non-responders had significantly higher expression of this protein in the initial tumor samples (Figure 1O). Representative examples of IHC detection of evaluated protein markers in the initial samples for responders and non-responders are also provided (Figure 3).

The expression patterns of DDX39A, HMGA1, HOXC9, and NF1 proteins in the post-induction samples were very similar to those in the initial samples, as described above (Figures 2A–L). The only apparent difference was found for PBX1: both the proportion of PBX1-positive cells as well as the IR and subsequently immunoscore values were reduced (Figures 2M–O).

**Figure 4:**
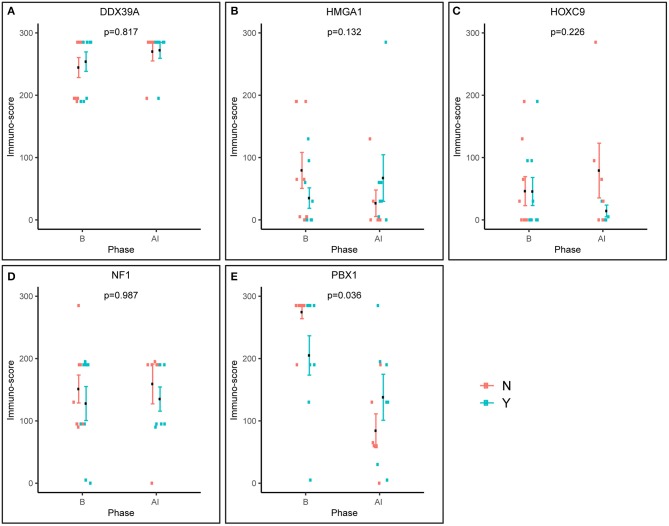
Pre-posttreatment differences in immunoscores between responders and non-responders as evaluated using factor ANOVA. Phase: B, baseline (initial samples); AI, after induction (post-induction samples). N, non-responder; Y, responder.

Furthermore, we also analyzed changes in the expression of these putative markers before and after induction chemotherapy according the response to treatment (Figure 4). Interestingly, the expression pattern of PBX1 changed inversely, and this change was also statistically significant (Figure 4E). A similar inverse expression pattern was also observed for HMGA1 (Figure 4B), but this trend was not significant.

### Analysis of Expression Patterns of Evaluated Proteins in Relation to the Survival Probability

**Figure 5:**
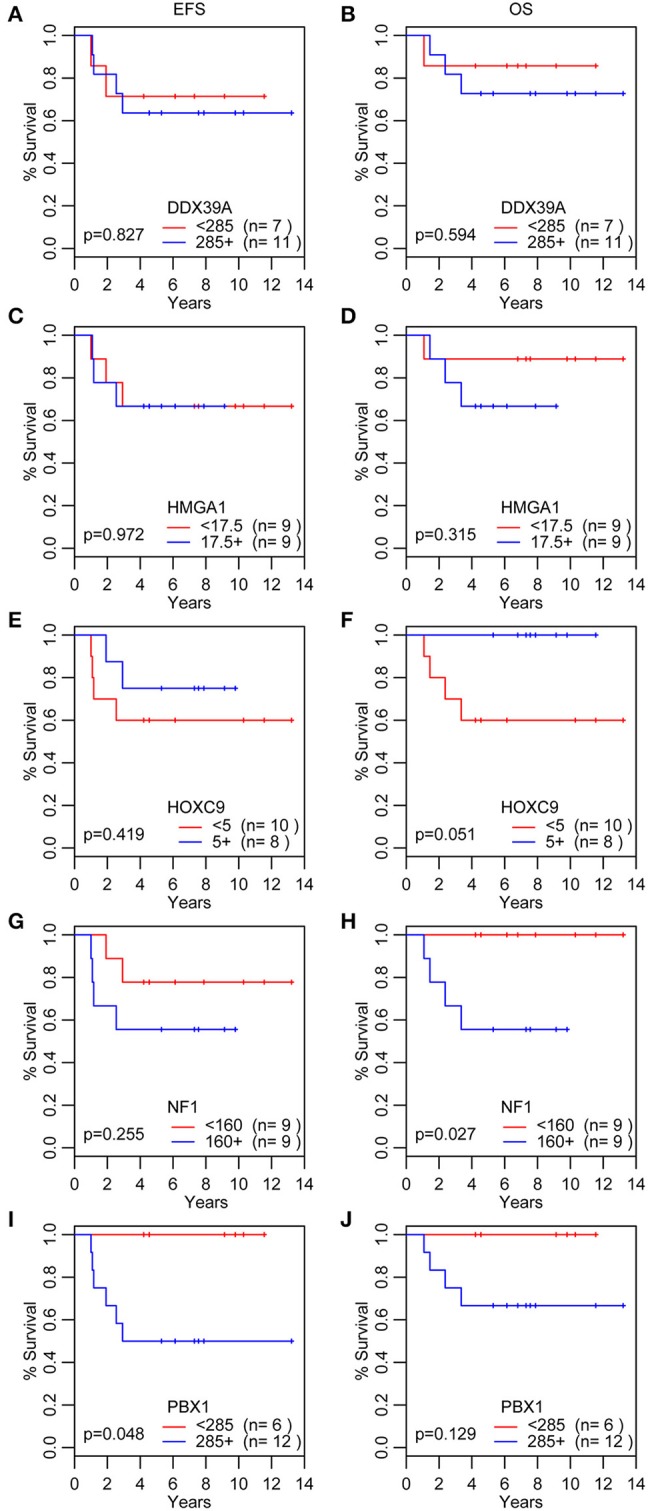
Analyses of survival probability. Kaplan-Meier Q20 curves stratified by the median values of the immunoscore for each particular protein marker. A red line indicates low expression; a blue line indicates the high expression. OS, overall survival **(B,D,F,H,J)**; EFS, event-free survival **(A,C,E,G,I)**.

In addition to the analysis of their possible predictive values, we also performed a detailed evaluation of the expression of these five proteins in relation to the probability of OS and EFS (Figure 5). High expression of NF1 (Figures 5G,H) was significantly related to reduced OS (*p* = 0.027). Similarly, high expression of PBX1 (Figures 5I,J) was significantly related to reduced EFS (*p* = 0.048) and the same—although statistically insignificant—trend was also found for OS. In contrast, low HOXC9 expression (Figures 5E,F) is apparently associated with reduced OS; however, this difference remained insignificant (*p* = 0.051). The results of 3- and 5-year Kaplan–Meier survival estimates with 95% confidence limits in parentheses are summarized in Table 4.

**Table 4:** Overview of 3- and 5-year Kaplan–Meier survival estimates with 95% confidence limits in parentheses.

**Parameter**	**Group**	***N***	**3-year survival (CI) %**	**5-year survival (CI) %**
**EFS**				
Score DDX39A	<285	7	71.4 (44.7–100)%	71.4 (44.7–100)%
	285+	11	63.6 (40.7–99.5)%	63.6 (40.7–99.5)%
Score HMGA1	<17.5	9	66.7 (42–100)%	66.7 (42–100)%
	17.5+	9	66.7 (42-100)%	66.7 (42–100)%
Score HOXC9	<5	10	60 (36.2–99.5)%	60 (36.2–99.5)%
	5+	8	75 (50.3–100)%	75 (50.3–100)%
Score NF1	<160	9	77.8 (54.9–100)%	77.8 (54.9–100)%
	160+	9	55.6 (31–99.7)%	55.6 (31–99.7)%
Score PBX1	<285	6	NA	NA
	285+	12	50 (28.4–88)%	50 (28.4–88)%
**OS**				
Score DDX39A	<285	7	85.7 (63.3–100)%	85.7 (63.3–100)%
	285+	11	81.8 (61.9–100)%	72.7 (50.6–100)%
Score HMGA1	<17.5	9	88.9 (70.6–100)%	88.9 (70.6–100)%
	17.5+	9	77.8 (54.9–100)%	66.7 (42–100)%
Score HOXC9	<5	10	70 (46.7–100)%	60 (36.2–99.5)%
	5+	8	NA	NA
Score NF1	<160	9	NA	NA
	160+	9	66.7 (42–100)%	55.6 (31–99.7)%
Score PBX1	<285	6	NA	NA
	285+	12	75 (54.1–100)%	66.7 (44.7–99.5)%

*Each score parameter was dichotomized based on the median value of the respective parameter (immunoscore). Cut-off values are indicated for each parameter. Survival cannot be estimated for groups with no events (indicated as NA). EFS, event-free survival, OS, overall survival*.

### Analysis of Expression Patterns of Evaluated Proteins and Survival Probability in Relation to the MYCN Status

Finally, we also perform the detailed analysis of the expression patterns among these five proteins in relation to the MYCN status: both in the initial (Supplementary Figure 2) and post-induction (Supplementary Figure 3) samples; no significant difference was found. Surprisingly, the MYCN amplification was not related to reduced OS or EFS (Supplementary Figure 4) in our cohort of patients.

## Discussion

Although all five of these proteins were previously reported as related to the prognosis or to the resistance/sensitivity to retinoids in NBL, our results apparently show that their usefulness as predictive markers in “real-life scenario” is limited. This discrepancy with previously published studies can be caused by several important factors that should be considered during the interpretation of our results. First, our study compared five putative markers that were previously analyzed separately by different research groups. Second, our comparative analysis was performed using FFPE tumor samples, not using cell lines or frozen samples; this biological model allowed us to compare the actual amount of the protein in question in real tumor tissue. Third, we analyzed both samples taken during initial biopsies and samples taken from the same patients after multimodal induction chemotherapy; this was a key new approach for evaluating the possible use of analyzed proteins as predictive biomarkers in NBL. Finally, our experimental design based on the homogenous cohort of patients suffering solely from high-risk NBL was focused both on the prediction of the clinical response to multiagent chemotherapy and the estimation of survival probability using Kaplan–Meier analysis in relation to the same set of clinical data. In the next paragraphs, we will discuss these markers one by one in light of our findings in comparison with the previously published results.

ATP-dependent RNA helicase DDX39A, also known as URH49, is a paralog of DDX39B helicase with 90% amino acid identity (18). In NBL, its expression was reported as an independent unfavorable prognostic factor when analyzed in primary tumor samples using IHC. Nevertheless, this cohort of patients was not fully comparable to ours because the samples included in this study were taken from patients with NBL of unknown risk categories and evaluated according to MYCN status (19). Thus, the same and relatively high levels of DDX39A in both initial (Figures 1A,B) and post-induction (Figures 2A,B) biopsies independent of the clinical outcome (responders vs. non-responders), as observed in our study, are not in direct contradiction and can be explained by different experimental designs. In other words, high levels of DDX39A in high-risk NBL are not surprising *per se* because patients with this category of NBL have a worse prognosis when compared with patients with other NBL risk categories. Consequently, the high median value of the immunoscore used as cutoff for this analysis according to our unified methodology is apparently not suitable for discrimination of these patients with high-risk NBL into different survival probability categories. Similar to the published findings (19), high levels of DDX39 were associated with poor prognosis in gastrointestinal stromal tumors (20). In contrast, low levels of DDX39 were reported as a marker of poor prognosis for bladder carcinoma (21) and colorectal carcinoma (22).

The HOXC9 protein belongs to the homeobox (HOX) family of transcription factors, members of which play an important role in the mediation of retinoid action during the development of the nervous system (12). HOXC9 was reported as a key regulator in the induced differentiation of NBL cells (23, 24). Our data showed no significant change in HOXC9 expression in relation to the response to induction therapy in either initial (Figures 1G,H) or post-induction (Figures 2G,H) biopsies. Unfortunately, no comparable data regarding HOXC9 and the therapeutic outcome have been published thus far. Nevertheless, high levels of HOXC9 were identified as markers associated with a better prognosis of survival in three different datasets obtained from NBL patients (14) and our results (Figures 5E,F) are in full accordance with these findings. Similar results were also previously reported for glioblastoma (25) and breast carcinoma (26).

A key role of neurofibromin 1 (NF1) within a cell is to downregulate activated RAS proteins, which results in the deactivation of RAS/MEK and PI3K/AKT signaling pathways (27). In NBL cells, NF1 is able to control a response to treatment with retinoids through RAS/MEK signaling because this cascade suppresses the expression of ZNF423 protein functioning as a RAR/RXR coactivator. Moreover, the combined expression status of NF1 and ZNF423 proteins was identified as a powerful prognostic marker in NBL: low levels of both of these proteins were associated with the worst prognosis for NBL patients, while high levels of expression of both proteins were related to the best progression-free interval (11). Despite these findings, other studies on the possible role of NF1 in tumorigenesis have indicated that expression of NF1 can be considered a negative prognostic factor in several cancer types (28). Thus, although our data showed no differences in NF1 expression between responders and non-responders, in both initial and post-induction tumor samples, it should be noted that high proportions of NF1-positive cells were detected in the majority of examined samples regardless of the response category or biopsy status (Figures 1J, 2J). Moreover, we found that high expression of NF1 in terms of immunoscore (Figures 5G,H) was significantly related to reduced OS (*p* = 0.027). As all the patients in our cohort were diagnosed with high-risk NBL, all of our results on NF1 correspond to the hypothesis on the relationship between NF1 overexpression associated with aggressive tumor behavior. Similar findings were recently published for colorectal carcinoma (29).

The HMGA subfamily of high-mobility-group (HMG) proteins consists of several members that serve as transcription factors directly binding to DNA or that regulate the expression of target genes via protein–protein interactions (30). Treatment with retinoids can change the expression of HMGA1, and these changes are closely related to MYCN status (30, 31). Although there are no data on the possible relationship between HMGA1 expression and clinical outcome in patients suffering from NBL, published studies on other cancer types suggest that HMGA1 overexpression is associated with aggressive tumor behavior and poor prognosis: such findings were reported for breast carcinoma (32–34), pancreatic adenocarcinoma (35), esophageal squamous cell carcinoma (36), non-small cell lung cancer (37), and uveal melanomas (38). In contrast, such results were not confirmed by other research groups for gastric cancer (39) and non-small cell lung cancer (40). Our findings showed slightly higher HMGA1 expression in initial biopsies taken from non-responders, as well as an inverse expression pattern in post-induction biopsies (Figure 4B), but these results and, similarly, the results of the survival analysis (Figures 5C,D) were not statistically significant. Nevertheless, such trends in expression—although non-significant—are in accordance with recent knowledge on the importance of HMGA1 as a “master regulator” in tumorigenesis and its association with tumor aggressiveness (32, 34, 38).

The most interesting of our results concerns pre-B-cell leukemia homeobox-1 (PBX1) protein. This molecule is a member of the three-amino-acid loop extension TALE family of atypical homeodomain proteins with characteristic three-residue insertion in the first helix of the homeodomain. PBX1 protein forms heterodimeric transcription complexes by interacting with other homeodomain-containing nuclear proteins, such as HOX and MEIS-1. PBX1 is involved in a variety of biological processes, including cell differentiation and tumorigenesis (41, 42). Very recently, PBX1 has been considered a group of pioneering factors that are able to initiate cell fate changes (43). PBX1 was identified as a critical component in NBL differentiation, and this is unique among the three-amino-acid loop extension family proteins. In NBL cell lines treated with 13-cis retinoic acid, PBX1 expression was induced only in sensitive cell lines, and reduced PBX1 levels led to an aggressive growth phenotype and resistance to 13-cis retinoic acid. In the same study, it was also demonstrated that PBX1 expression correlates with histological NBL subtypes, with the highest expression in benign ganglioneuromas and the lowest expression in high-risk NBL (13). In contrast, our study revealed that the highest levels of PBX1 in tumor tissue are associated with poor response to induction chemotherapy, whereas PBX1 levels were decreased in non-responders (Figures 1M–O, 2M–O). More interestingly, the PBX1 expression pattern was inverted after induction chemotherapy, and this change was statistically significant (Figure 4E). Furthermore, the survival analysis clearly demonstrated that high levels of PBX1 in tumor tissue are significantly associated with a worse clinical prognosis (Figures 5I,J). The explanation of such different results can be found in the heterogeneity of samples and methods used in the previous study mentioned above (13): (i) the evaluation of PBX1 prognostic value was performed by reverse transcription quantitative PCR, not by the IHC method used in our study, and (ii) their samples were taken from tumors of various risk categories, which is in contrast to our samples acquired solely from high-risk NBL.

To summarize, our study provides new insight into the usefulness of the biomarkers described above for the prediction of responses to multiagent chemotherapy in patients suffering from high-risk NBL. Although these molecules were still considered prognostic biomarkers, our results showed that the expression patterns of only two of those biomarkers HMGA1 and especially PBX1 differ before and after induction chemotherapy. Moreover, high levels of PBX1 are significantly associated with a poor response to induction chemotherapy and with worse clinical outcome in our cohort of patients. Similarly, we found a significant relationship between high levels of NF1 and worse survival probability in terms of OS. We argue that, although the reported statistical significances were not corrected, we consider the findings robust and significant. First, we observed a good agreement of the results (namely, for PBX1) across different analyzes such as the immunoscores and the survival. Second, there is an issue of the risk of overcorrection, which was already shortly described in *Material and Methods*. Owing to relatively small number of patients involved in our study, this interesting finding should be verified in the independent larger case series. As all of these molecules were also reported to be involved in the treatment of NBL cells with retinoids, it would be helpful to elucidate this issue. Because it is very difficult to collect a large set of paired NBL samples before and after treatment with retinoids, this study cannot answer the question about the usefulness of these markers for predicting the response of patients to retinoids. Nevertheless, our already published study on this topic using a set of primary NBL cell lines confirmed the association of low levels of PBX1 with the sensitivity to retinoids (15).

## Data Availability Statement

All datasets generated for this study are included in the article/Supplementary Material.

## Ethics Statement

The studies involving human participants were reviewed and approved by The Research Ethics Committee of the School of Science, Masaryk University (Brno, Czech Republic). Written informed consent was obtained from the minor(s)' legal guardian/next of kin for the publication of any potentially identifiable images or data included in this article.

## Author Contributions

RV, PC, and JS designed the study. PM and JS provided tumor samples and relevant clinical data. MJ performed immunohistochemical analyses. MK performed statistical analyses. RV composed the manuscript. PC and VD participated in data analyses and manuscript preparation. All authors reviewed and approved the final version of the manuscript.

### Conflict of Interest

The authors declare that the research was conducted in the absence of any commercial or financial relationships that could be construed as a potential conflict of interest.
