# Supplementary material for: Comparative Analysis of Putative Prognostic and Predictive Markers in Neuroblastomas: High Expression of PBX1 Is Associated With a Poor Response to Induction Therapy
Source: Front Oncol. 2019 Nov 15;9:1221. doi: 10.3389/fonc.2019.01221 (PMC6872531; doi:10.3389/fonc.2019.01221)
Supplement: Supplementary file 1 [file Data_Sheet_1.PDF]

Supplementary Material

1 Supplementary Figures

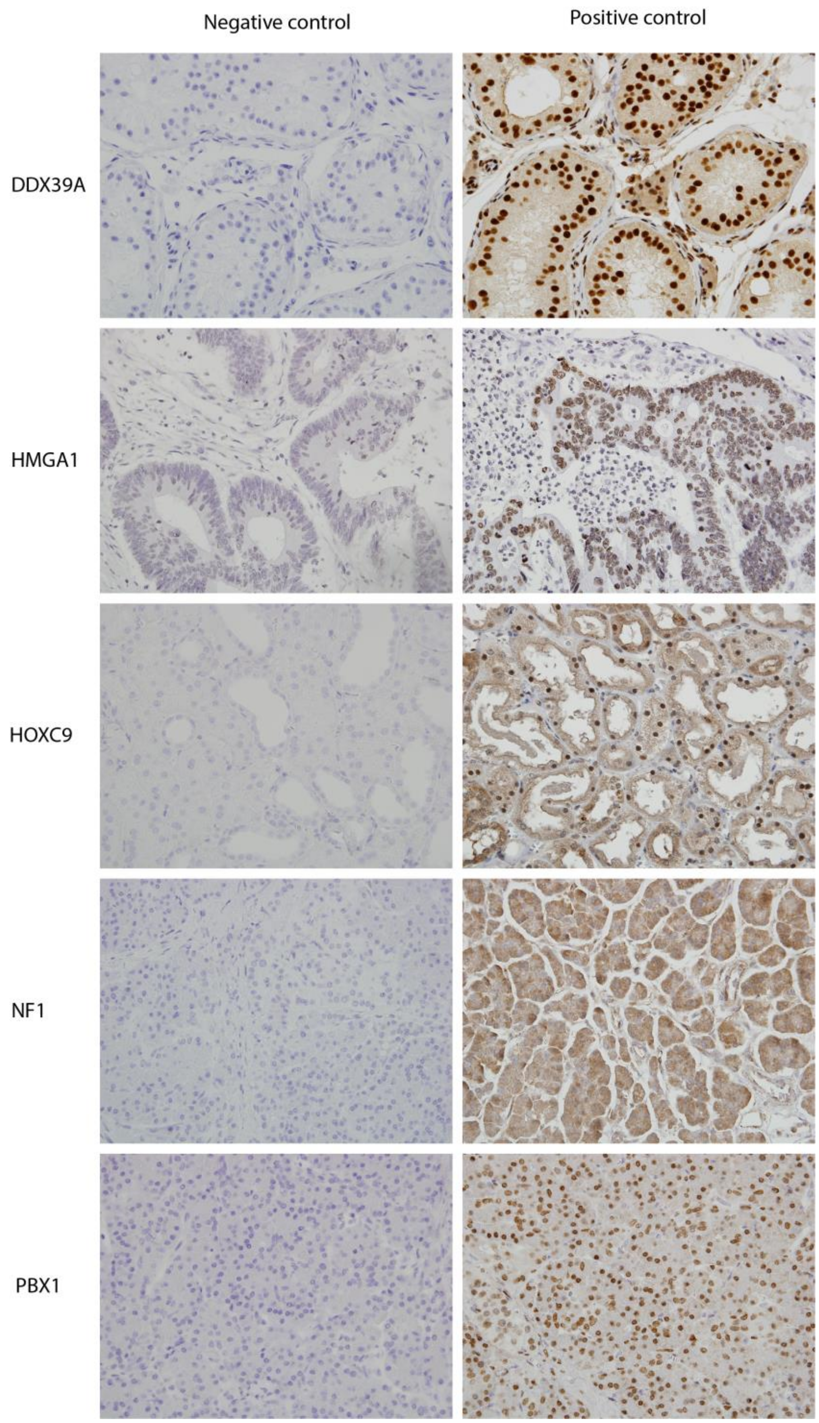

Supplementary Figure 1. Negative and positive controls for IHC staining. Negative control samples processed without primary antibodies and representative expressions of the evaluated proteins in positive control samples. Original magnification, 400×.

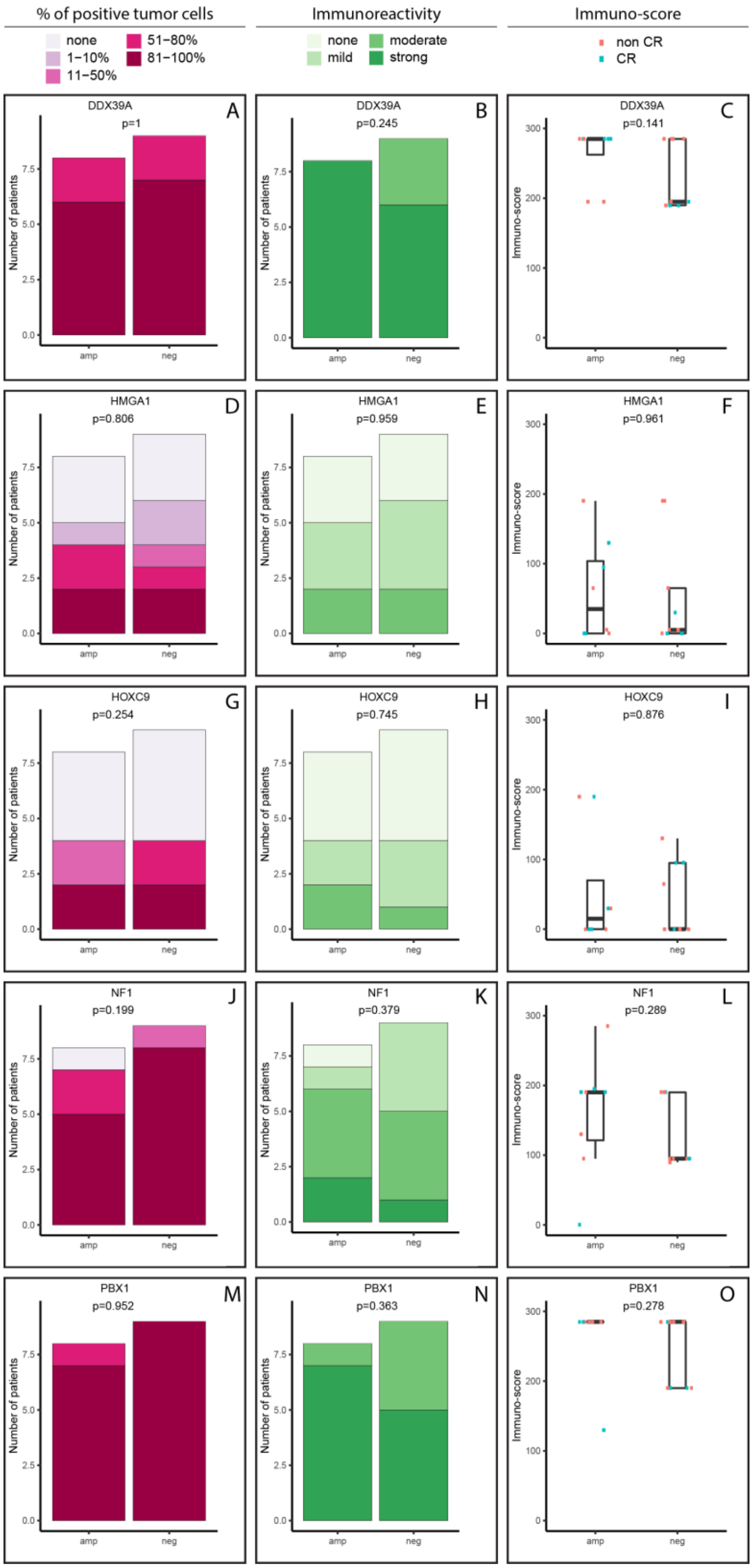

Supplementary Figure 2. Comparative IHC analysis of DDX39A, HMGA1, HOXC9, NF1, and PBX1 in the initial samples in relation to the MYCN status. Immuno-scores were calculated for individual antigens by multiplying the median percentage category of positive cells by their respective immunoreactivity. MYCN status: amp, amplified; neg, not amplified. CR, complete remission.

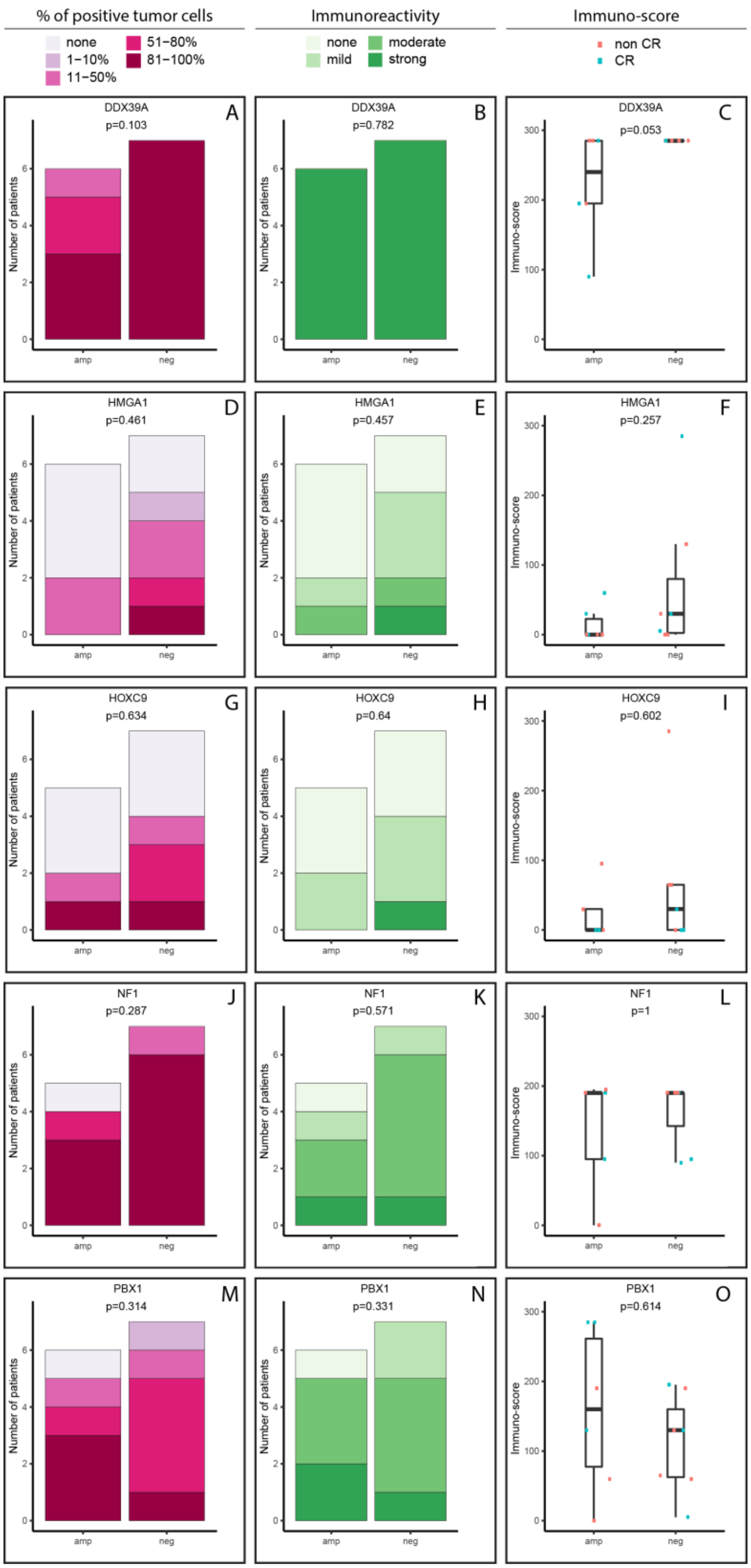

Supplementary Figure 3. Comparative IHC analysis of DDX39A, HMGA1, HOXC9, NF1, and PBX1 in the postinduction samples in relation to the MYCN status. Immuno-scores were calculated for individual antigens by multiplying the median percentage category of positive cells by their respective immunoreactivity. MYCN status: amp, amplified; neg, not amplified. CR, complete remission.

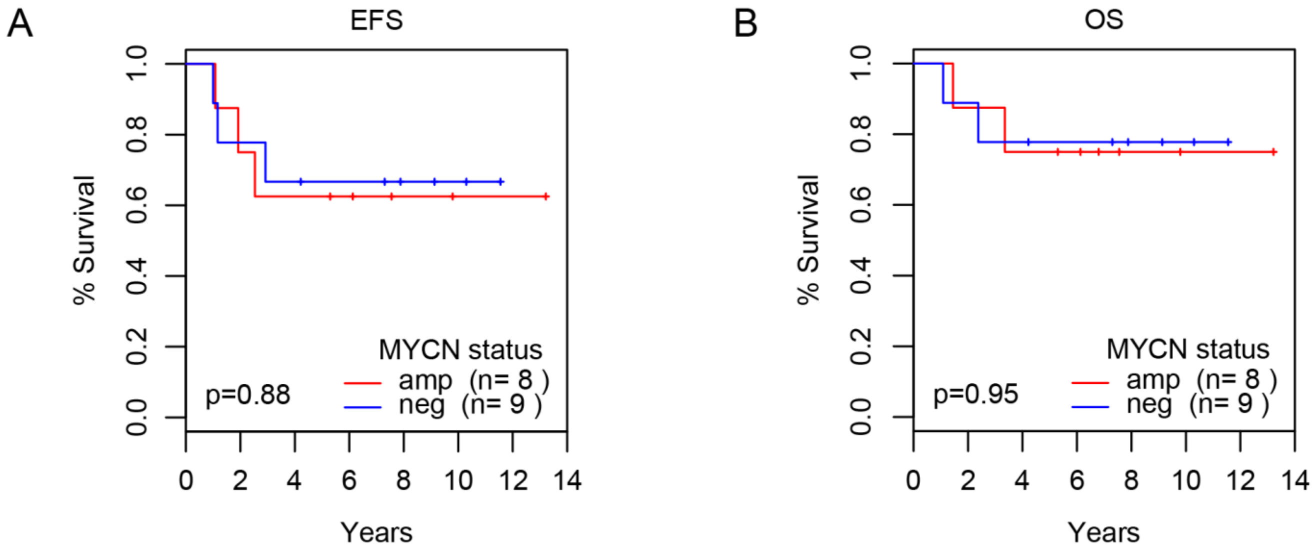

Supplementary Figure 4. Analyses of survival probability in relation to the MYCN status. Kaplan-Meier curves stratified by the different MYCN status. A red line indicates the amplified MYCN; a blue line indicates the non-amplified MYCN. OS, overall survival; EFS, event-free survival.
